# Comparative analyses of two primate species diverged by more than 60 million years show different rates but similar distribution of genome-wide UV repair events

**DOI:** 10.1186/s12864-021-07898-3

**Published:** 2021-08-06

**Authors:** Umit Akkose, Veysel Ogulcan Kaya, Laura Lindsey-Boltz, Zeynep Karagoz, Adam D. Brown, Peter A. Larsen, Anne D. Yoder, Aziz Sancar, Ogun Adebali

**Affiliations:** 1 Molecular Biology, Genetics & Bioengineering Program, Faculty of Engineering and Natural Sciences, Sabanci University, 34956 Istanbul, Turkey; 2 Department of Biochemistry and Biophysics, School of Medicine, University of North Carolina at Chapel Hill, Chapel Hill, North Carolina 27599 USA; 3 Department of Pharmacology and Cancer Biology, Duke University, Durham, North Carolina 27708 USA; 4 Department of Biology, Duke University, Durham, North Carolina 27708 USA; 5 Present Address: Department of Veterinary and Biomedical Sciences, University of Minnesota, St. Paul, MN 55112 USA

**Keywords:** Nucleotide excision repair, UV damage, XR-seq, Mouse Lemur, (6–4)PP, CPD, Primate

## Abstract

**Background:**

Nucleotide excision repair is the primary DNA repair mechanism that removes bulky DNA adducts such as UV-induced pyrimidine dimers. Correspondingly, genome-wide mapping of nucleotide excision repair with eXcision Repair sequencing (XR-seq), provides comprehensive profiling of DNA damage repair. A number of XR-seq experiments at a variety of conditions for different damage types revealed heterogenous repair in the human genome. Although human repair profiles were extensively studied, how repair maps vary between primates is yet to be investigated. Here, we characterized the genome-wide UV-induced damage repair in gray mouse lemur, *Microcebus murinus*, in comparison to human.

**Results:**

We derived fibroblast cell lines from mouse lemur, exposed them to UV irradiation, and analyzed the repair events genome-wide using the XR-seq protocol. Mouse lemur repair profiles were analyzed in comparison to the equivalent human fibroblast datasets. We found that overall UV sensitivity, repair efficiency, and transcription-coupled repair levels differ between the two primates. Despite this, comparative analysis of human and mouse lemur fibroblasts revealed that genome-wide repair profiles of the homologous regions are highly correlated, and this correlation is stronger for highly expressed genes. With the inclusion of an additional XR-seq sample derived from another human cell line in the analysis, we found that fibroblasts of the two primates repair UV-induced DNA lesions in a more similar pattern than two distinct human cell lines do.

**Conclusion:**

Our results suggest that mouse lemurs and humans, and possibly primates in general, share a homologous repair mechanism as well as genomic variance distribution, albeit with their variable repair efficiency. This result also emphasizes the deep homologies of individual tissue types across the eukaryotic phylogeny.

**Supplementary Information:**

The online version contains supplementary material available at 10.1186/s12864-021-07898-3.

## Background

Nucleotide excision repair is an essential mechanism to remove bulky DNA adducts, including UV-induced DNA lesions [1]. As in other repair systems, excision repair starts with damage recognition. Two subpathways based on damage recognition lead to two repair mechanisms: global repair (GR) and transcription-coupled repair (TCR). GR is active throughout the genome, whereas TCR is only active on the transcribed strands as it is initiated by damage recognition through stalled RNA polymerase II (RNAPII). To date, many techniques have been developed to detect DNA damage and repair [2]. The approach of some methodologies has been NGS-based, which allows answering genome-wide questions. To reveal genome-wide excision repair dynamics, heterogeneity and associations, eXcsion Repair sequencing (XR-seq) was developed. The XR-seq technique directly measures the repair events by capturing excised DNA oligomers containing the lesion. It was found that TCR is more efficient, particularly for slowly-repaired DNA lesions. For instance, among UV photoproducts 6–4 pyrimidine-pyrimidone ([6–4]PP) and cyclobutane pyrimidine dimer (CPD), CPD is more prone to TCR as GR less efficiently repairs it. Although DNA lesions might preferentially form at certain local sites in the genome, the overall heterogeneity of repair is mainly due to uneven repair efficiency throughout the genome [3, 4]. Genome-wide heterogenous repair distribution is mostly caused by transcription and chromatin structure [5–8].

To date, genome-wide repair maps were generated for model organisms, including *Escherichia coli* [9, 10], *Saccharomyces cerevisiae* [11], *Drosophila melanogaster* [12], *Arabidopsis thaliana* [13], *Mus musculus* [14], and *Homo sapiens* [3, 5]. TCR presence was verified for each of these species. For eukaryotic genomes, the consistent finding was the efficient repair in open chromatin regions. Heterochromatin regions were found to be repaired at later time points. Human repair profiles were extensively studied with respect to damage formation and chromatin states. Whether regions in the human genome that are efficiently repaired are organism-specific is yet to be investigated.

To study whether repair patterns are unique to the organism of interest, we aimed to compare human and a deeply diverged non-human primate. Gray mouse lemur (*Microcebus murinus*) stands out as a promising model organism candidate because of its small body size, short gestation time (2 months) and fast sexual maturation (6–8 months) [15, 16]. A near chromosome level reference genome for the gray mouse lemur was recently sequenced and assembled [17]. With no surprise, it was shown that mouse lemur and human orthologs share ~ 91% identity. Although a robust genome assembly is available, we lack an in-depth understanding of this species’ genomic features such as epigenetic maps, transcriptomes and methylomes.

Given that mouse lemurs and humans last shared a common ancestor at the base of the primate clade [18], the same cell types from human and mouse lemur should behave similarly in response to DNA damage as a reflection of their deep homology. With this motivation, we carried out a comparative study between these two primates to understand similarities and differences between their repair profiles. We derived primary fibroblasts from mouse lemur and immortalized them. We performed survival assays in response to UV stress, immunoslot blot assays and in vivo excision assays for both cell lines. From mouse lemur fibroblasts, we obtained transcriptomes, exposed cells to UV and performed XR-seq. XR-seq captured the excised oligomers as repair products for two main UV-induced damage types: (6–4)PP and CPD. We compared lemur and human fibroblast XR-seq datasets for their genomic repair distribution.

## Results

### Excised oligomer characteristics

**Fig. 1:**
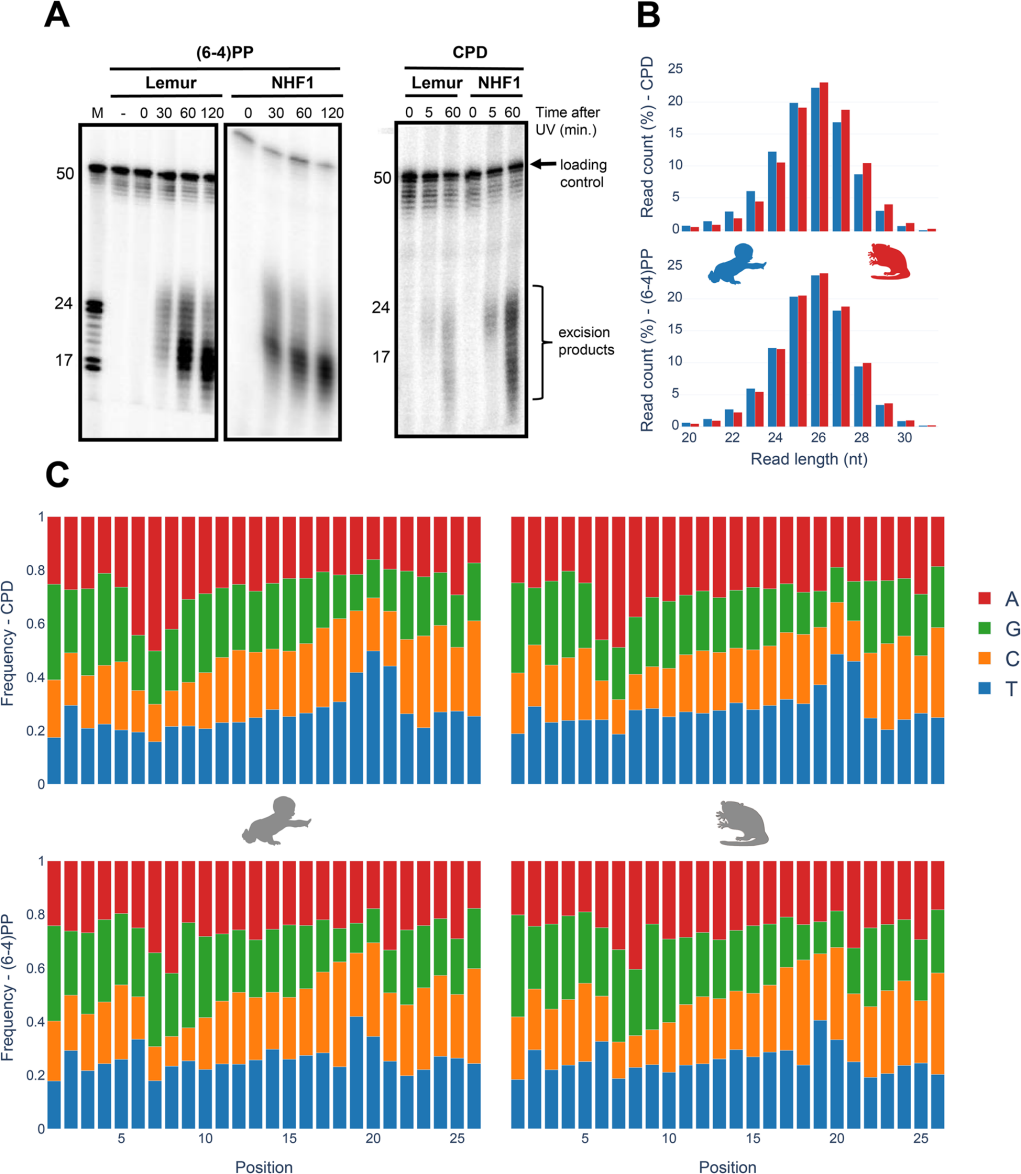
Characteristics of excised oligomers upon repair of UV-induced damages. (**A**) In vivo excision assay for (6–4)PP and CPD are shown. (**B**) XR-seq read length distribution of excised oligomers for CPD (top) and (6–4)PP (bottom). Human and mouse lemur are shown in blue and red, respectively. (**C**) Nucleotide frequency in the predominant oligomer (26 nt) for each primate and damage type. Representative data from replicate 1

The in vivo excision assay was used to analyze the excised oligomers containing UV-induced lesions (Fig. 1A). Excised oligomers were captured for the two distinct damage types with specific anti-damage antibodies. The oligomer length distribution varies from 16 to 30 nt. Two intense bands were observed, which indicate primary and degraded excised oligomers. The gel images show that the primary excised oligomer lengths vary between 23 and 25 nt. Through the time course, gel images reflect more intense secondary products, indicating higher levels of degraded excised oligomers at later time points. For normal human fibroblasts 1 (NHF1) and mouse lemur fibroblasts, this trend is similar. The human and lemur experiments were not conducted together and thus, the band intensities differ. The spike-in 50-mer internal control was used for quantification of the excision products between experiments.

Nucleotide excision repair in human, *S. cerevisiae* and *E. coli* was shown to yield primary excised oligomers predominantly in lengths of 27, 24 and 13 nucleotides, respectively [9, 11, 19–21]. Mouse lemur XR-seq resulted in oligomer length distribution identical to humans (Fig. 1B). For both CPD and (6–4)PP, nucleotide excision repair machinery incised 26 nt oligomer as the predominant product. A small variance in oligomer lengths between the in vivo excision assay and XR-seq might be due to the exonuclease activity on the excised oligomers in the vivo excision assay. Because of the TFIIH coimmunoprecipitation in the excision repair sequencing technique, XR-seq primary products are not yet degraded, possibly because of TFIIH protection of the excised oligomers.

### Identical CPD and (6–4)PP nucleotide frequency between human and lemur

Nucleotide frequencies of the excised oligomers between human and mouse lemur are identical for both CPD and (6–4)PP (Fig. 1C). Pyrimidine enrichment was revealed at 19th, 20th and 21st nucleotides for CPD and (6–4)PP for both organisms, which is in agreement with the incision site at 6 to 8 nt fixed distance to the 3′ end of the DNA lesion. The nucleotide contents for CPD and (6–4)PP are different, as they were previously reported and discussed [5].

### UV repair efficiency of mouse lemur in comparison with human

**Fig. 2:**
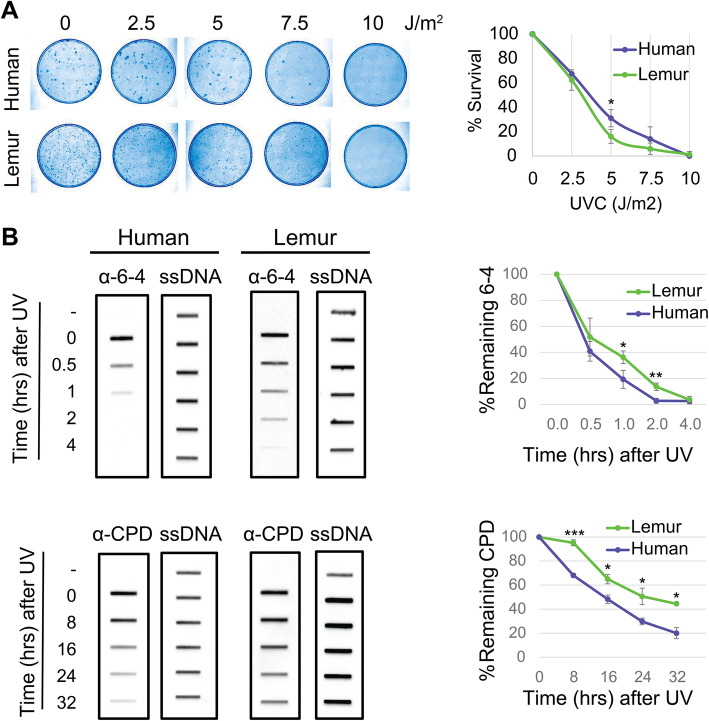
UV sensitivity and repair rates in human and lemur fibroblasts. (**A**) Clonogenic survival assay (left) and the quantified results (right). (**B**) Immunoslot blot repair assays to examine the repair kinetics of UV-induced DNA lesions for both organisms and each damage type (left). Remaining damage levels at each time point were quantified and plotted (right). All experiments were repeated three times, and graphed data are presented as (mean+/−SD). Tests (t-test) performed (H_0_:μ_human_ = μ_lemur_) for each dosage (**A**) and time points (**B**). * *P* ≤ 0.05; ** *P* ≤ 0.01; *** *P* ≤ 0.001; rest *P* > 0.05

To identify the overall repair efficiency upon UV irradiation, we performed a colony survival assay. We seeded 300 cells and grew them for 16 h. Cells were exposed to UV-C at 1 J/m2sec for variable time intervals to reach indicated doses ranging from 2.5 to 10 J/m2. We counted cells for each dose and plotted survival curves for lemur and human fibroblasts (Fig. 2A). Based on the survival curves of the cell lines used in this study, we infer that mouse lemur fibroblasts are more sensitive to UV relative to human cells.

We then performed immunoslot blot assays to measure the DNA repair rates (Fig. 2B). Cells were exposed to UV-C at 10 J/m2. To separate UV-induced DNA lesions, we used specific antibodies for CPD and (6–4)PP. We quantified the remaining damage signal for each damage type for both species. We observe a difference between rates of repair of CPD and (6–4)PP, as has been previously described, which is due to several reasons including differences in damage recognition and differences in global repair versus transcription coupled repair discussed further below. Both (6–4)PP and CPD repair appeared to be more efficient in human cells compared to mouse lemur. Human (6–4)PP repair was completed in 2 h whereas in lemurs, it took 4 h to complete (6–4)PP repair. 20% of CPDs were still unrepaired for human cells 32 h after UV irradiation. Unrepaired CPD ratio was more than 40% for mouse lemur cells. Survival and immonoslot blot assays are in harmony and they suggest mouse lemur cells have relatively low repair rates compared to NHF1 cells.

### Prominent transcription-coupled repair in mouse lemur

Nucleotide excision repair comprises two sub-pathways, each of which distinctly acts on damage recognition. GR is the genome-wide mechanism that is active in any region in the genome, although its efficiency depends on the type of damage as well as chromatin factors. On the other hand, TCR depends on the RNAPII stalled at the lesion. Stalled RNAPII recruits transcription-coupled repair factor CSB to enhance repair in the transcribed strand of the genes. DNA lesions in non-transcribed strands are subject to global repair. A major rate-limiting step of nucleotide excision repair is damage recognition. Therefore, minor helix-distorting lesions such as CPDs are recognized more efficiently when they stall elongating RNAPII, and thus genes have asymmetrical repair between two strands; the transcribed strand (TS) is repaired more efficiently compared to the nontranscribed strand (NTS) [22, 23].

**Fig. 3:**
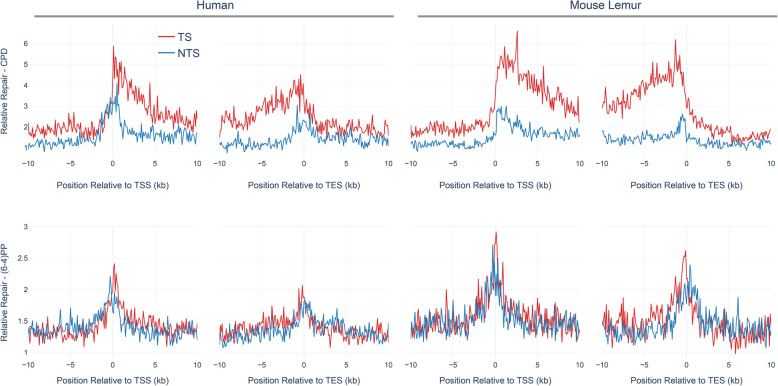
Transcription-coupled repair in mouse lemur and human fibroblasts. Average profiles of CPD XR-seq (top) and (6–4)PP XR-seq (bottom) over 5277 and 3366 annotated genes are plotted for human (left) and mouse lemur (right), respectively. Transcription start sites (TSS) and transcription end sites (TES) were retrieved from GTF (Gene Transfer Format) files for the two genomes. Transcribed (red) and nontranscribed (blue) strand repair are shown in the downstream and upstream of TSS and TES, respectively. 10 kb upstream and downstream of TSS and TES were divided into 100 bp windows. XR-seq reads aligned to each bin were normalized to RPKM (reads per kilobase per million mapped reads). The XR-seq RPKM values were normalized by the shuffled RPKM values derived by mapped reads aligned to random genomic sites. Only replicate 1 is shown. TS/NTS median values for TSS downstream for CPD are 1.76 and 2.31 for human and mouse lemur, respectively

Global repair for (6–4)PP is much faster than for CPD [7, 24]. TCR removes a minor fraction of the (6–4)PPs, and thus strand asymmetry between TS and NTS (TS/NTS ratio) is much weaker for (6–4)PP compared to TS-favored strand asymmetry of CPD repair [7]. CPDs, on the other hand, are harder to be recognized by global repair, and therefore it takes a longer time to remove CPDs from the genome. For this reason, at early time points (such as 1 h after UV irradiation), we observe a strong TCR effect, which is indicated by the asymmetrical repair between TS and NTS. Here, we compared the genic strand asymmetry between human and mouse lemur (Fig. 3). We retrieved all annotated protein-coding genes from both genomes. We removed the genes that are closer to each other with less than 20 kb in order to remove the signal that might have a “canceling-out” effect. We used 5277 and 3366 annotated genes for human and mouse lemur, respectively (see methods). We performed a meta-analysis where we aligned all transcription start and transcription end sites and calculated the RPKM (reads per kilobase per million mapped reads) values. Interestingly, mouse lemur fibroblasts exhibited stronger TCR profiles compared to human cell lines at 1 h after CPD formation (TS/NTS Mann-Whitney U test *p* = 4.3*10–21). The higher TCR profile of lemur fibroblasts might be caused by the lower global repair efficiency relative to human fibroblasts (see discussion).

### Conserved repair rates between homologous regions of two primates

**Fig. 4:**
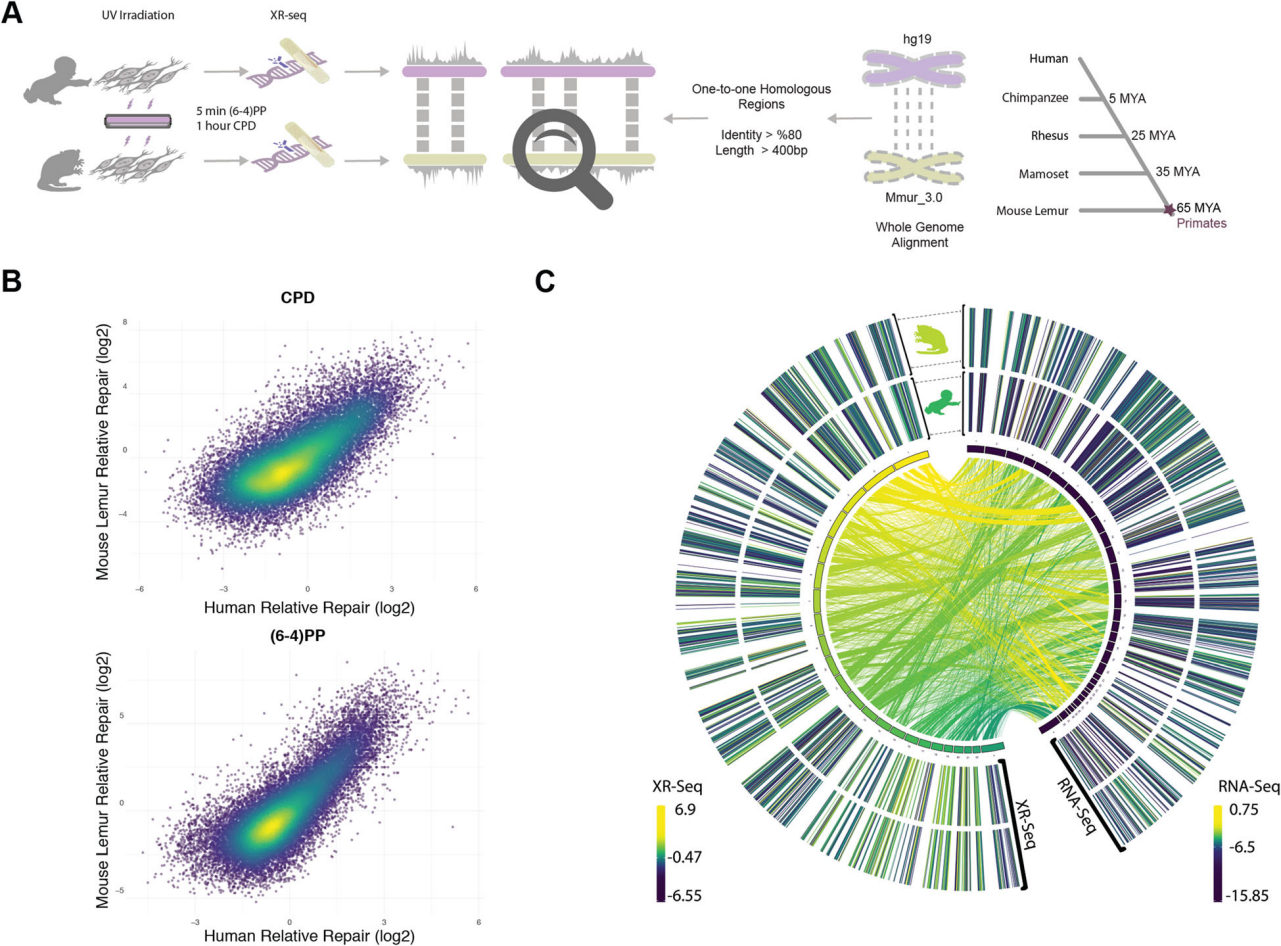
Comparative analysis methodology and correlated repair rates between human and mouse lemur. **A**) Approach to identify orthologous regions between human and mouse lemur (see methods for details). **B**) Scatter plots showing the normalized repair levels between two organisms. Relative repair was calculated by normalizing the repair signal by the simulated XR-seq reads to eliminate the sequence context bias. **C**) Mapped orthologous regions with repair and transcription profiles mapped at left and right outer rings, respectively. Inner circle represents the human (left) and mouse lemur (right) chromosomes. Inner lines connecting orthologous regions are colored based on the human chromosome color scale. Repair and transcription values (outer rings) for genomic regions that have no evident ortholog (based on the criteria in A) are not shown

In order to compare and contrast the genome-wide repair profiles between human and mouse lemur, we retrieved the homologous genomic regions between the organisms (Fig. 4A). We aligned the two genomes and kept the regions that have at least 80% identity. Due to the low coverage of the XR-seq samples we removed the aligned segments that are shorter than 400 bases to eliminate the random effect due to scarce repair events. Lineage-specific duplications (or deletions) result in paralogs that can be co-orthologs to one locus in the other genome. This situation is known as the “one-to-multiple” orthology relationship. As we cannot be sure which one of the multiple paralogs in one genome is the “true” ortholog of a region in the other one, we filtered out one-to-multiple kinds of homologous relationships and kept one-to-one homologs only.

1.0 and 1.3% of the genomes were taken into account with one-to-one homologs for human and mouse lemur, respectively. 4–5% and 2–3% of the XR-seq reads mapped to those regions for CPD and (6–4)PP, respectively. The reason why the mapping rate of CPD is higher than (6–4)PP could be due to its slower repair rate, as slowly repaired CPDs are more prone to the effects of chromatin. Because we applied stringent criteria to retrieve the homologous regions with at least 80% identity, they are likely to be more open compared to other regions in the genome. Therefore, more CPD repair events aligned to those regions relative to (6–4)PP reads.

We investigated the correlation between the repair profiles of the two species. Because excised oligomers have a certain nucleotide bias due to the damage site, we normalized the true repair signal (XR-seq) by the simulated XR-seq reads. Simulated reads have the nucleotide content of the true XR-seq reads (Fig. S1), however, they are randomly retrieved from the homologous regions. With this approach, we eliminated the potential bias of the nucleotide content that creates a pseudo-correlation. Although there is a clear difference in the relative TCR rates between two species (Fig. 3), homologous regions of two genomes exhibited a strong repair rate correlation; R values for (6–4)PP and CPD are 0.80 and 0.72 (*p* = 0), respectively (Fig. 4B). Not only repair but also transcription levels between the two species are correlated (Fig. 4C). The similar repair and transcription profiles between two cell lines are independent of their chromosomal locations. Chromosomal homologous region associations are dispersed, as previously suggested [17].

### Repair profile correlation is associated with gene expression

**Fig. 5:**
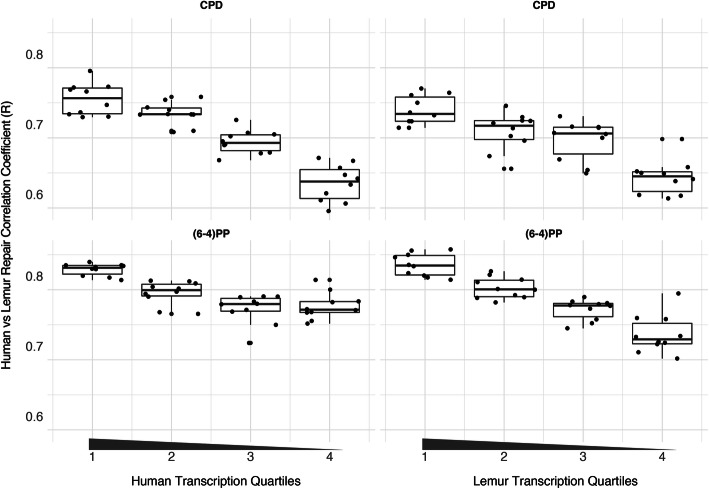
Repair rate consistency in correlation with gene expression. The transcription levels of the orthologous regions were divided into quartiles separately for human and lemur. Out of the quartiles, 10 bootstrapped subsamples were retrieved, and for those regions repair correlation was analyzed. Correlation coefficient (*R* values) are represented on the y-axis; quartiles are on the x-axis

To test whether gene expression is a factor associated with the correlation of repair between these two highly-diverged species of primates, we examined the correlation strength and gene transcription levels. We prepared RNA-seq data sets for mouse lemur fibroblasts and obtained RNA-seq datasets for human fibroblasts from a publicly available database (see methods). The strength of the correlation between the two repair profiles was found to be correlated with transcription levels (Fig. 5). Highly expressed regions had better repair correlation between the two species (Figs. S2; S3; S4; S5). The data did not show a correlation between the identity levels and repair correlation between the two species, which suggests that gene expression levels rather than the degree of homology might be an important factor.

### The same cell type from two primates have more similar repair profiles relative to two different cell types from the same organism

**Fig. 6:**
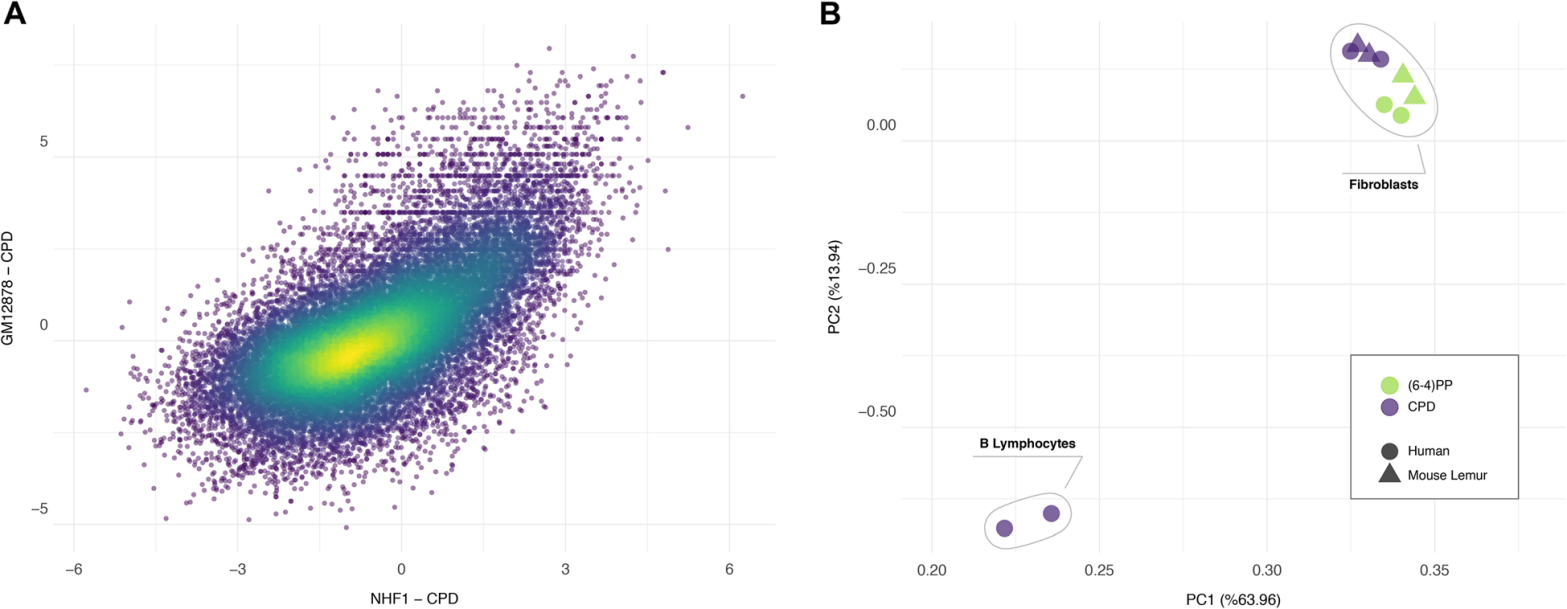
Cell type-based consistency of repair profiles between two primates. **A**) Scatter plot or normalized repair levels between two human cell lines, GM18787 and NHF1, show correlation (*R* = 0.65, *p* = 0). **B**) Principle component analysis of 10 XR-seq samples

We cannot dismiss the possibility that the correlation of repair profiles between two primates could simply be due to the heterogenous mappability of the genomes. Such an association makes sense only when we measure the similarity of repair patterns relative to another dataset. For this reason, we used another human cell type, GM12878 - B-Lymphocytes to ask whether the repair profile of human fibroblasts is closer to lemur fibroblasts or human lymphocytes. We performed XR-seq for CPD as it yields relatively high genome-wide heterogeneity compared to (6–4)PP. Although repair profiles between human GM12878 and NHF1 are also correlated (Fig. 6A), this correlation is not as strong as the one between human and mouse lemur fibroblasts. Additionally, human and mouse lemur fibroblasts clustered together in the PCA plot (Fig. 6B). Within fibroblasts, biological replicates grouped together. More interestingly, (6–4)PP and CPD samples formed distinct groups, each of which has human and mouse lemur samples in the subclusters. The two replicates of CPD XR-seq samples of human lymphocytes (GM12878) clustered as an outgroup. When we applied PCA differentially to genes and intergenic regions, we observed similar clustering (Fig. S6). This result suggests that not only transcriptional but also epigenetic profiles between human and mouse lemur fibroblasts are similar to yield correlated TCR (genes, Fig. S6A) and global repair (intergenic regions Fig. S6B), respectively. On the other hand, even though derived from the same organism, completely different human cell lines have a considerable variation with respect to repair patterns. It is interesting to note that although TCR profiles between two primates are quite different from each other, they share high similarity in repair profiles.

## Discussion

XR-seq methodology has become prominent in genome-wide profiling of nucleotide excision repair at single-nucleotide resolution. This technique has been applied to a variety of model organisms, from humans to bacteria. Although genome-level understanding of human repair behavior is relatively well established in recent years, the associations between closely related primates with respect to UV damage response and genome-wide repair distribution were entirely lacking. With this pilot study, we established the differences and similarities between mouse lemur and human using a pair of similarly derived fibroblast cell lines. First, we observed repair efficiency differences between the two genomes of the two major types of DNA damage induced by UV. Like in humans, (6–4)PP was much more quickly completed compared to CPD in mouse lemur. Both (6–4)PP and CPD repair efficiencies were slower in mouse lemur fibroblasts relative to human fibroblasts. This observation is in agreement with the survival assays, which show that mouse lemur cell lines were more sensitive to UV, which contains both damage types we studied. Although this comparison is limited to only two species, and further analysis would be required to make a more definitive conclusion, we postulate that the differences could result from differing repair enzyme activity between the species because of differences in fur which protects from UV exposure or due to the differences in diurnal and nocturnal lifestyles of the two species.

Interestingly, we also observed that TCR was relatively more efficient in mouse lemurs compared to humans, particularly for CPD repair. TCR of (6–4)PP was not prominent, as was previously shown, and our immunoslot blot assays indicate that (6–4)PP repair is fast and therefore, repaired mostly by GR rather than TCR. We observed similar TS and NTS repair signal for (6–4)PP in genic regions, also suggesting that GR is the primary subpathway to repair (6–4)PP. A striking fact is an inverse correlation between TCR, which is measured by the TS/NTS repair signal, and overall repair efficiencies. Mouse lemurs with more efficient TCR fall behind in total repair compared to the human. A possible explanation for this observation is that global repair is the primary determinant of the repair efficiency. XR-seq yields the entire nucleotide excision repair events with no distinction between TCR and GR. For this reason, GR and TCR efficiencies derived through XR-seq are relative to each other. Like the relativity in any RNA-seq, which cannot measure the total transcription levels, XR-seq also gives the relative distribution of the repair events. Therefore, measured “TCR efficiency” actually corresponds to “GR deficiency” in the XR-seq profile. In other words, when the damage is not efficiently recognized by the GR pathway, TCR takes over the recognition and removes DNA lesions in TS. With this study, previously observed high TCR levels in *Arabidopsis thaliana* [13] and mouse [14] now also suggest relatively poor GR efficiency in those organisms.

Human evolution has been under investigation since the time of Charles Darwin. Comparative analyses between humans and other animals have revealed both shared and human-specific features. In the era of molecular biology, it has been possible to identify molecular features as they are shared between humans and their relatives. Primates are an extremely diverse group comprised of species that range in body size from 30 to 80 g (mouse lemurs) to 150 kg (African apes). Mouse lemurs stand out as the world’s smallest living primate. Given detailed knowledge of primate phylogeny and divergence times [25], we know that the most recent common ancestor (MRCA) of humans and mouse lemurs is MRCA of all living primate species and that this MRCA likely arose near the Cretaceous-Paleogene (K-Pg) boundary 65 mya. Thus, we can reasonably conclude that features that are shared between humans and mouse lemurs are homologous and characteristic of the primate clade.

We have a limited knowledge of the epigenetic architecture of mouse lemurs as we far lack chromatin data. Prior to this study, it was unknown whether repair profiles would be similar in the comparison of two primates species. Our work revealed that human and mouse lemur fibroblasts are significantly similar with respect to their preference on which genomic region to repair first. This bias is likely to be caused by the chromatin structure, as previously shown in other organisms [8]. Damage distribution is known to be homogenous at large genomic scales [3]. Therefore, it is safe to assume that repair preference is the major factor for XR-seq heterogeneity. The repair profiles of human and mouse lemur fibroblasts are much more similar to each other than the ones between two distinct human cell lines. Moreover, (6–4)PP and CPD repair profiles are more similar to themselves, suggesting similar repair dynamics between two organisms. Further studies on mouse lemur epigenetics will possibly explain the most differentiated genomic repair levels in mouse lemurs. Better understanding mouse lemur genomics, as well as other species within the strepsirrhine clade, will provide additional insight into human evolution, repair and mutagenesis.

## Conclusions

We demonstrated that genome-wide UV repair profiles between human and and mouse lemur are strongly correlated. Our results shed light onto deep homologies of same tissue types in primates by suggesting similar epigenetic architecture between them. We believe our results on varying repair rates between the two species will guide follow-up studies investigating the evolutionary effects of day time activity and UV avoidance on differential nucleotide excision repair rates.

## Methods

### Cell lines and reagents

#### Establishment of the immortalized mouse lemur fibroblast cell line

Primary fibroblasts were established using methods outlined in Larsen and Harris et al. [17]. For the current study, we used an immortalized version of these primary mouse lemur fibroblasts. This cell line was generated by transfecting passage six primary cells at approximately 60% confluency with 5 μg of human hTERT (hTERT) plasmid DNA per well of a 6-well plate using Lipofectamine® 2000 diluted in Opti-MEM® medium. Twenty-four hours post-transfection, cells were placed under selection using G418 sulfate at a concentration of 300 μg/mL for two weeks. Selected cells were then plated into a 96-well plate and remained under G418 sulfate maintenance at a concentration of 100 μg/mL to establish single cell colonies. Approximately 2 weeks post-transfection, colonies were observed in 19 of the 96 wells and these cells underwent subsequent propagation and expansion.

#### Human cell lines

Normal human fibroblasts (NHF1) were telomerase-immortalized and obtained from W.K. Kaufmann as previously described [5, 26]. All cell lines were grown in Dulbecco’s modified Eagle’s medium (DMEM) containing 10% fetal bovine serum (FBS) in a 5% CO2 incubater at 37 °C. Human lymphocyte GM12878 was purchased from Coriell.

### Clonogenic survival assay

Cells were plated at 300 cells per 10 cm plate, grown overnight, and then UV irradiated under a GE germicidal lamp (254-nm UVC light) at 1 J/m^2^sec for the indicated dose. The cells were cultured for seven days after irradiation, rinsed with phosphate-buffered saline (PBS), treated with 75% methanol and 25% acetic acid solution for 10 min to fix the cells, and then treated for 30 min with a solution of 0.5% crystal violet in 25% methanol to stain the cells before the plates were rinsed with water and allowed to air dry. Images of the plates were captured with a Bio-Rad Chemi-Doc XRS+ Molecular Imager, the stained colonies were counted, and the fraction surviving on the UV irradiated plates was determined relative to the number of colonies on the plates not treated. The experiment was repeated three times with each condition in triplicate.

### Immunoslot blot analysis

The measurement of UV photoproduct repair in genomic DNA was performed as previously described [27]. Cells were irradiated with UVC at a dose of 10 J/m^2^ and then incubated for the indicated times. A QIAamp DNA Mini kit was used to isolate genomic DNA, and 250 ng of DNA was immobilized on a nitrocellulose membrane using a Bio-Rad immunoslot blot apparatus and then incubated under vacuum for 90 min at 80 °C. After blocking the blots in 5% milk, they were probed with anti-(6–4)PP (Cosmo Bio 64 M-2 cat#NM-DND-001) or anti-CPD (Cosmo Bio TDM-2 cat#NM-DND-002) antibodies as indicated. The signal from anti-mouse horseradish peroxidase IgG secondary antibody (GE Healthcare catalog no. NA931V) was detected with Bio-Rad Clarity ECL Substrate using a Bio-Rad Chemi-Doc XRS+ Molecular Imager. To ensure equal DNA loading, the blots were re-blotted with anti-ssDNA antibody (Millipore MAB3034 clone 16–19). Experiments were performed three independent times, and representative results are presented.

### Excised oligonucleotide detection

Oligonucleoties excised by nucleotide excision repair was analyzed as described previously [19]. Cells grown in 15-cm plates to ~ 80% confluency, treated with 20 J/m^2^ of UVC, and then allowed to repair for the indicated times before harvesting. The cells were lysed with a modified Hirt procedure and the excised oligonucleotides were purified with either anti-(6–4)PP or anti-CPD antibodies. The UV photoproduct-containing oligos were 3′-end-labeled with terminal deoxynucleotidyl transferase (New England Biolabs) in the presence of [α-32P]-3′-deoxyadenosine 5′-triphosphate (cordycepin, Perkin Elmer). As an internal control, a 50-nucleotide oligomer (2.5 fmol) was included in the reactions. Oligonucleotides of known length were included as size markers on the urea-containing polyacrylamide gels. The gels were analyzed using a phosphorimager, and the experiments were repeated three times.

### XR-seq library preparation

Samples were processed for XR-seq, as previously described [28]. Cells grown in 15-cm plates to ~ 80% confluency were harvested either 5 min or 1 h after UVC irradiation with 20 J/m^2^, depending on the damage, (6–4)PP or CPD respectively, to be analyzed. Samples from four plates were pooled, lysed, and immunoprecipitated with anti-TFIIH antibodies (Santa Cruz Biotechnology, sc292 against p62 and sc293 against p89/XPB), and then processed for next generation sequencing.

### RNA-seq library preparation

Total RNA was isolated from one 10-cm plate of exponentially growing NHF1 or Lemur cells using Trizol (Invitrogen) and RNeasy Minis (Qiagen) following the manufacturer’s instructions. Novogene Co., Ltd. performed the library preparation and strand-specific paired-end sequencing (2 × 150 bp) on a HiSeq 4000 platform (Illumina). 

#### Human and lemur genome alignment

MUMmer version 3.23 [29] was used to align the grey mouse lemur genome (Mmur_3.0) to the human genome (hg19) with nucmer subprogram. The alignments were filtered based on criteria taking length and identity into account. Alignments were listed in “delta format” by default by Nucmer. For every reference (human) - query (lemur) pair, we kept the alignments which form the longest mutually consistent set. The filtering step was performed with delta-filter subprogram. Show-coords subprogram was used to display summary information such as position, percent identity and other features of each alignment, in Btab format with -B and -rclo arguments.

We used generateBED.R [30] to write homologous human-lemur regions in a bed file. Due to low genome coverage in XR-seq data sets, short regions might introduce random repair values. Since we were not interested in too short alignments, we filtered out alignments shorter than 400 bp while generating the bed file. Additionally, alignments with at percent similarity lower than 80 were filtered out of the list. The output was a BED file with each homologous region between two genomes.

This output file contained regions in either genome that align to multiple regions in the other genome. To remove such regions, we used, we used bedtools intersect [31] to intersect the file with itself. The command line we used was: bedtools intersect -wo -s -a humanOverlapsLemur_short_noFilter.bed -b humanOverlapsLemur_short_noFilter.bed > human_intersect.txt. We compared this intersected output file to the bed file generated previously using findDupAln.R [30] to exclude regions in both genomes that align to multiple regions in other genome. This resulted in a bed file of one-to-one homologous regions without multiple alignments for one region.

### XR-seq analysis

We trimmed 3′ adapter sequences (TGGAATTCTCGGGTGCCAAGGAACTCCAGTNNNNNNACGATCTCGTATGCCGTCTTCTGCTTG) using Cutadapt [32]. Bowtie2 version 2.3.4.1 [33] was used to align sequencing reads onto reference genomes with default parameters. The alignment in sam format was converted (with SAMtools [34]) to bam format, followed by conversion to bed format with BEDTools [31]. The resulting bed files were sorted and duplicate regions were removed. Command line we used was: sort -u -k1,1 -k2,2n -k3,3n ${SAMPLE}_cutadapt.bed >${SAMPLE}_cutadapt_sorted.bed.

### RNA-seq analysis

We aligned sequencing reads to the reference genomes using STAR Aligner 2.6.1a [35] with default parameters. The aligned reads were converted to bed format with bedtools bamtobed. The resulting bed files were sorted by coordinates and duplicate regions were removed. Command-line we used was: sort -u -k1,1 -k2,2n -k3,3n ${SAMPLE}.bed >${SAMPLE}_sorted.bed.

### XR-seq simulation

We evaluated the correlation of repair mechanisms with both simulated and real XR-seq datasets. Simulated datasets were generated for the overlapping regions between the grey mouse lemur genome and the human genome using ART simulator [36]. We used bedtools getfasta program with the bed file containing homologous regions between two organisms to generate a reference fasta file for ART simulator to produce synthetic reads. By default, ART simulator produces a fastq file with flat nucleotide distribution. For a better representation of XR-seq characteristics in the simulated dataset, we obtain nucleotide distribution frequencies of both species XR-seq reads. We applied a scoring function where each nucleotide in the simulated read was scored based on the frequency of that nucleotide to be in that position in the actual XR-seq dataset and obtained a total score for each simulated read. Accordingly, best scoring 10 million reads were selected for both species from the simulated dataset using filter_syn.go [30].

### Downstream analysis

To calculate nucleotide frequencies of excised oligomers, we first converted aligned Xr-seq reads to fasta format using bedtools getfasta. Using frequency.go [30] we calculated the nucleotide frequency of predominant oligomer (26 nt) for each primate and damage type.

Command line used to get excised oligomer lengths: awk ‘{print $3–$2}’ ${SAMPLE}_cutadapt_sorted.bed | sort -k1,1n | uniq -c | sed ‘s/\s\s*/ /g’ | awk ‘{print $2″\t’’$1}’.

Statistical analyses of immunoslot blot repair assays and clonogenic survival assay have been performed with Welch Two Sample t-test by using t-test.R [30].

For both genomes, all annotated protein-coding genes were retrieved and genes that are closer to each other with less than 20 kb were filtered out. We used 5277 and 3366 number of genes for human and mouse lemur, respectively. 10 kb upstream and downstream of TSS and TES were divided into 100 bp windows. XR-seq reads falling onto each bin were normalized to RPKM with tcr.py [30] Then XR-seq RPKM values were normalized by the RPKM values derived by the mapped reads onto random genomic sites which are prepared using bedtools shuffle for protein-coding genes that are not closer to each other with less than 20 kb. Statistical significance of TCR profiles was estimated using Mann-Whitney U-test [37]. Statistical analyses were performed using mann-w.py [30] with SciPy [38].

We used regions_rpkm.go [30] to calculate RPKM values of XR-seq, RNA-seq, and simulated XR-seq for each overlapping region. Regions with no XR-seq or RNA-seq reads were filtered out.

Circos plot for comprehensive summarization of all findings has been generated with circosPlot.R [30] by using R package circlize [39].

PCA plot has been generated from read counts with plotPCA.R [30], while applying variance stabilizing transformation as described in DESeq2 [40] vignette.

Plots were prepared using plotly and ggplot2 [41]. The manuscript was written using Manubot [42].

## Supplementary Information

**Additional file 1: Fig. S1**. Nucleotide content of simulated XR-seq pseudo-oligomers. **Fig. S2**. Human and mouse lemur (6–4)PP repair correlation in human transcription quartiles. **Fig. S3**. Human and mouse lemur (6–4)PP repair correlation in mouse lemur transcription quartiles. **Fig. S4**. Human and mouse lemur CPD repair correlation in human transcription quartiles. **Fig. S5**. Human and mouse lemur CPD repair correlation in mouse lemur transcription quartiles. **Fig. S6**. PCA plot for XR-seq reads mapped on the genic (**A**) and intergenic (**B**) regions.

## Data Availability

All raw and processed sequencing data generated in this study have been submitted to the NCBI Gene Expression Omnibus (GEO; https://www.ncbi.nlm.nih.gov/geo/) under accession number GSE145883. The code repository for the data analyses can be accessed at https://github.com/CompGenomeLab/lemurRepair.

## Abbreviations

GR: Global repair
TCR: Transcription-coupled repair
RNAPII: RNA polymerase II
XR-seq: excision repair sequencing
CPD: cyclobutane pyrimidine dimer
(6–4)PP: 6–4 pyrimidine-pyrimidone photo products
NHF1: normal human fibroblasts 1
TS: transcribed strand
NTS: non-transcribed strand
RPKM: reads per kilobase per million mapped reads
GEO: Gene Expression Omnibus
