# Supplementary material for: Comparative analyses of two primate species diverged by more than 60 million years show different rates but similar distribution of genome-wide UV repair events
Source: BMC Genomics. 2021 Aug 6;22:600. doi: 10.1186/s12864-021-07898-3 (PMC8349011; doi:10.1186/s12864-021-07898-3)
Supplement: Supplementary file 1 — Additional file 1: Fig. S1. Nucleotide content of simulated XR-seq pseudo-oligomers. Fig. S2. Human and mouse lemur (6–4)PP repair correlation in human transcription quartiles. Fig. S3. Human and mouse lemur (6–4)PP repair correlation in mouse lemur transcription quartiles. Fig. S4. Human and mouse lemur CPD repair correlation in human transcription quartiles. Fig. S5. Human and mouse lemur CPD repair correlation in mouse lemur transcription quartiles. Fig. S6. PCA plot for XR-seq reads mapped on the genic (A) and intergenic (B) regions. [file 12864_2021_7898_MOESM1_ESM.docx]

## Supplementary Materials


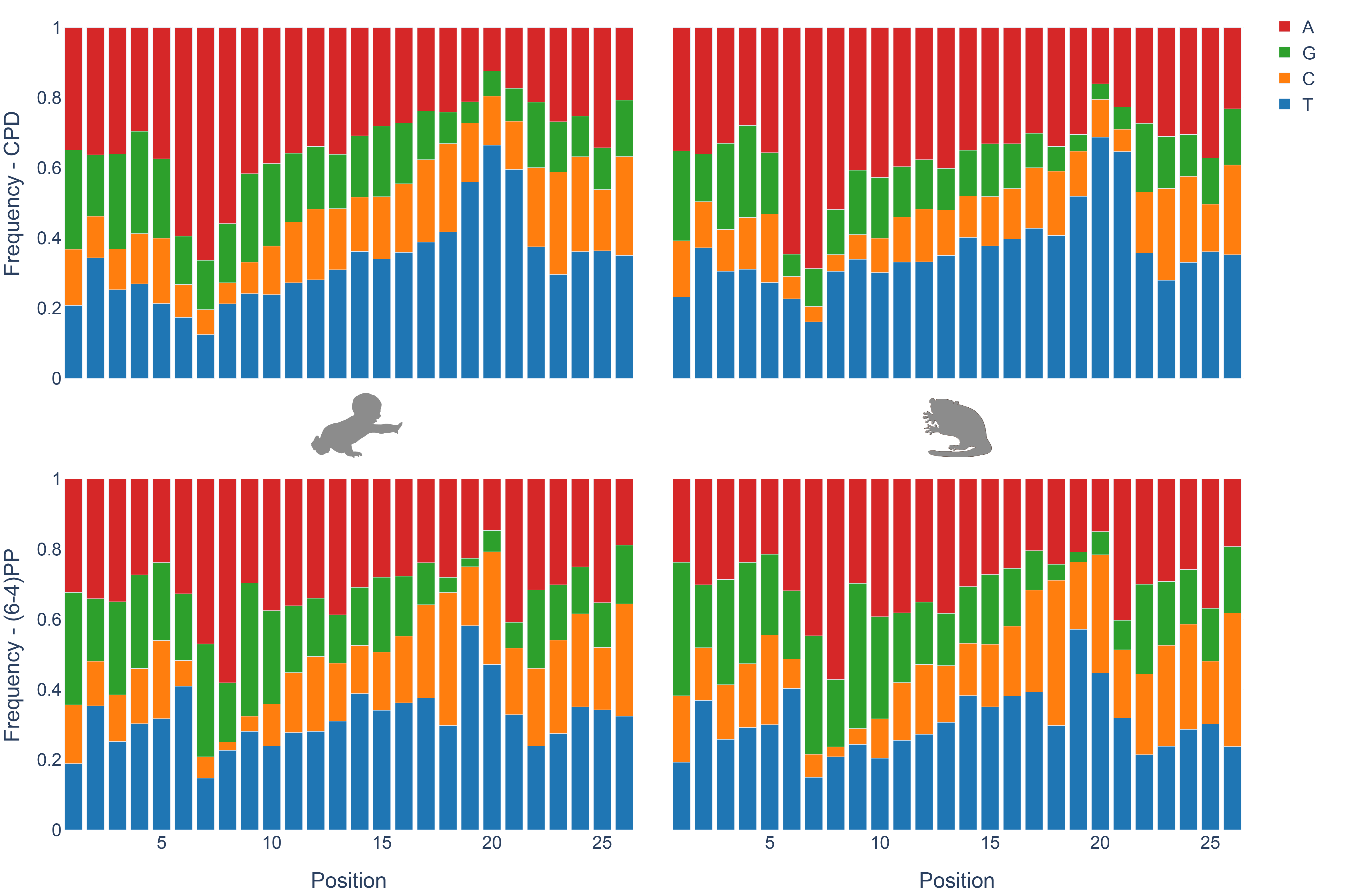


Figure S1: Nucleotide content of simulated XR-seq pseudo-oligomers.


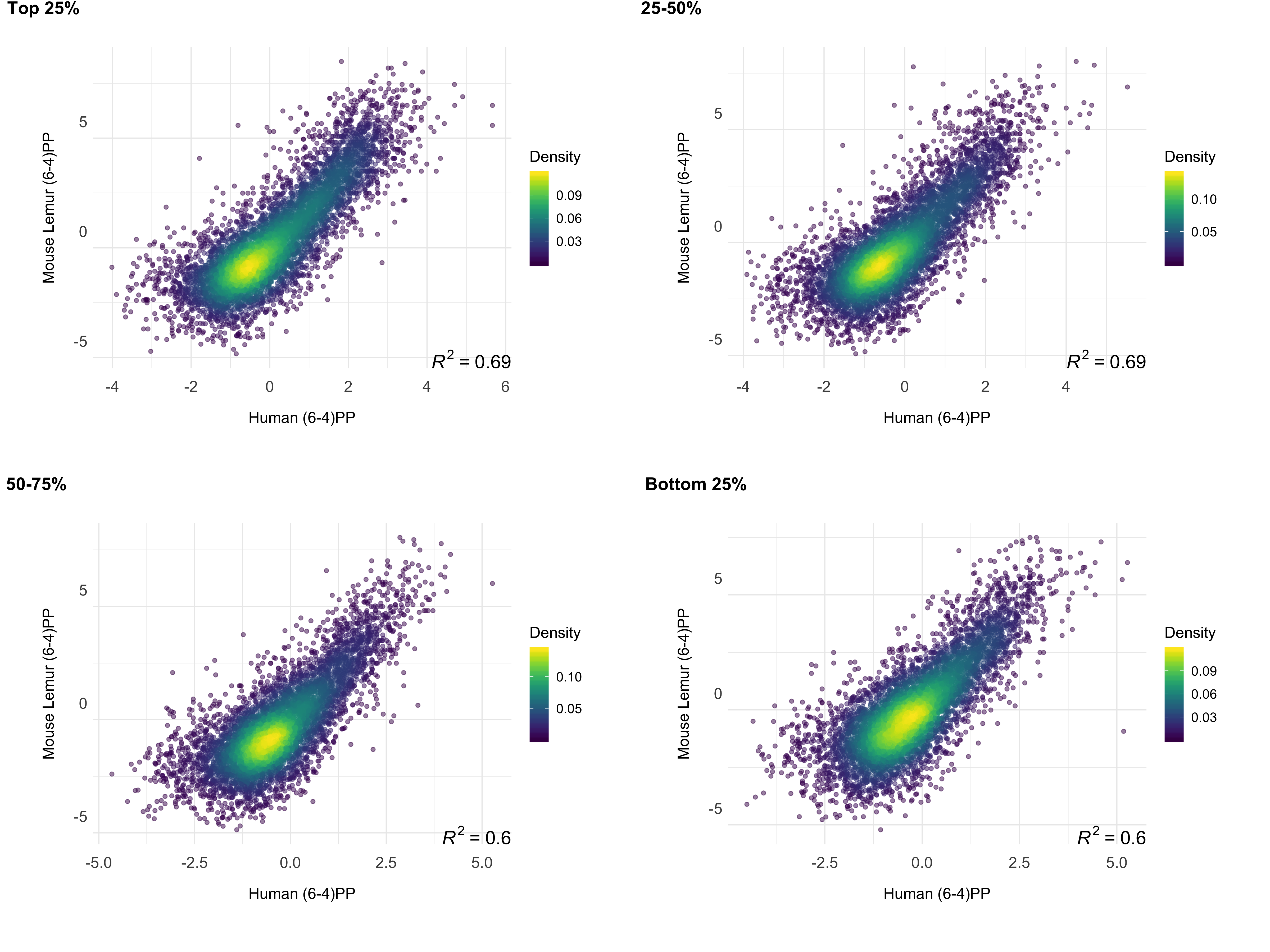


Figure S2: Human and mouse lemur (6-4)PP repair correlation in human transcription quartiles.


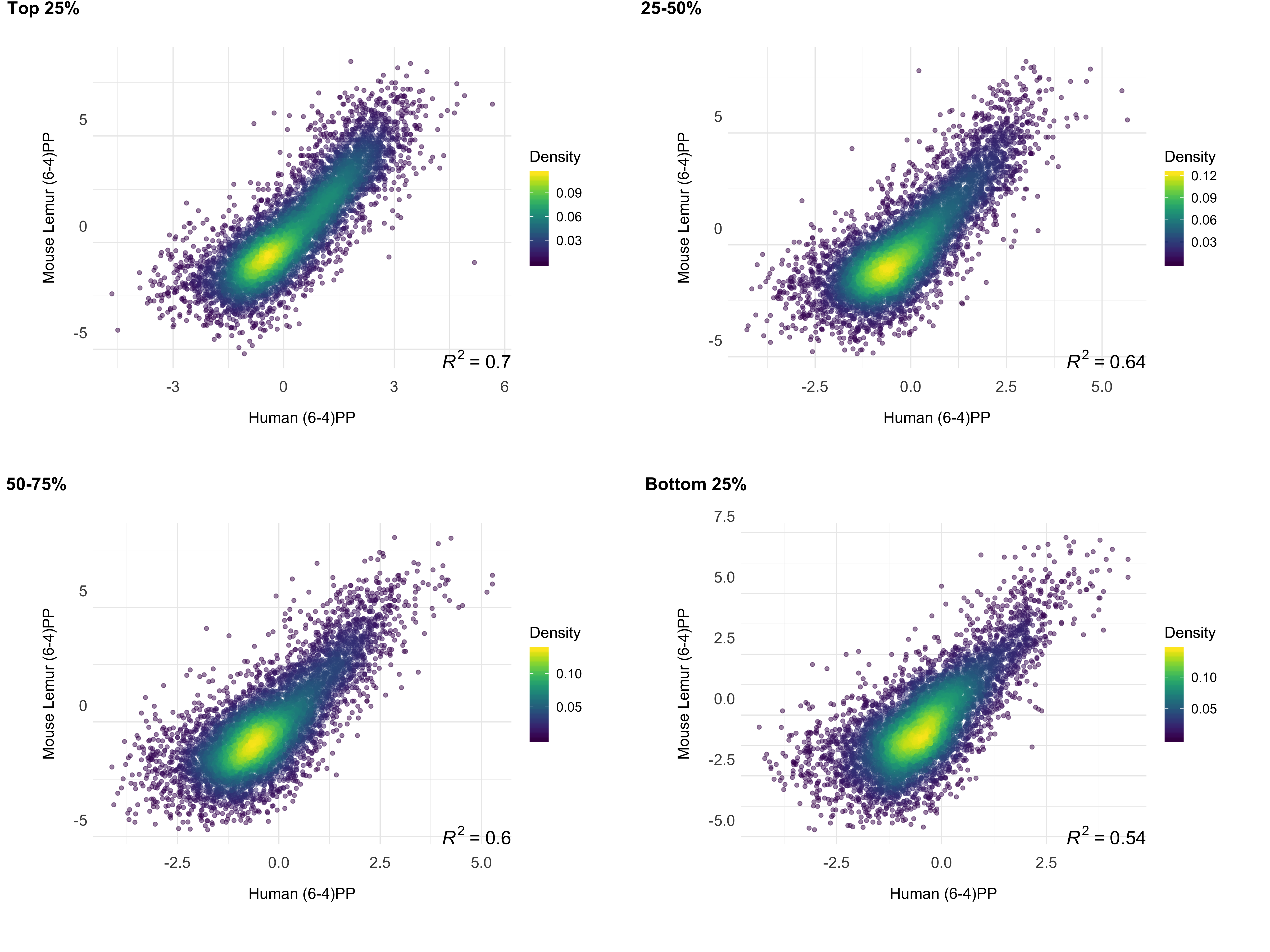


Figure S3: Human and mouse lemur (6-4)PP repair correlation in mouse lemur transcription quartiles.


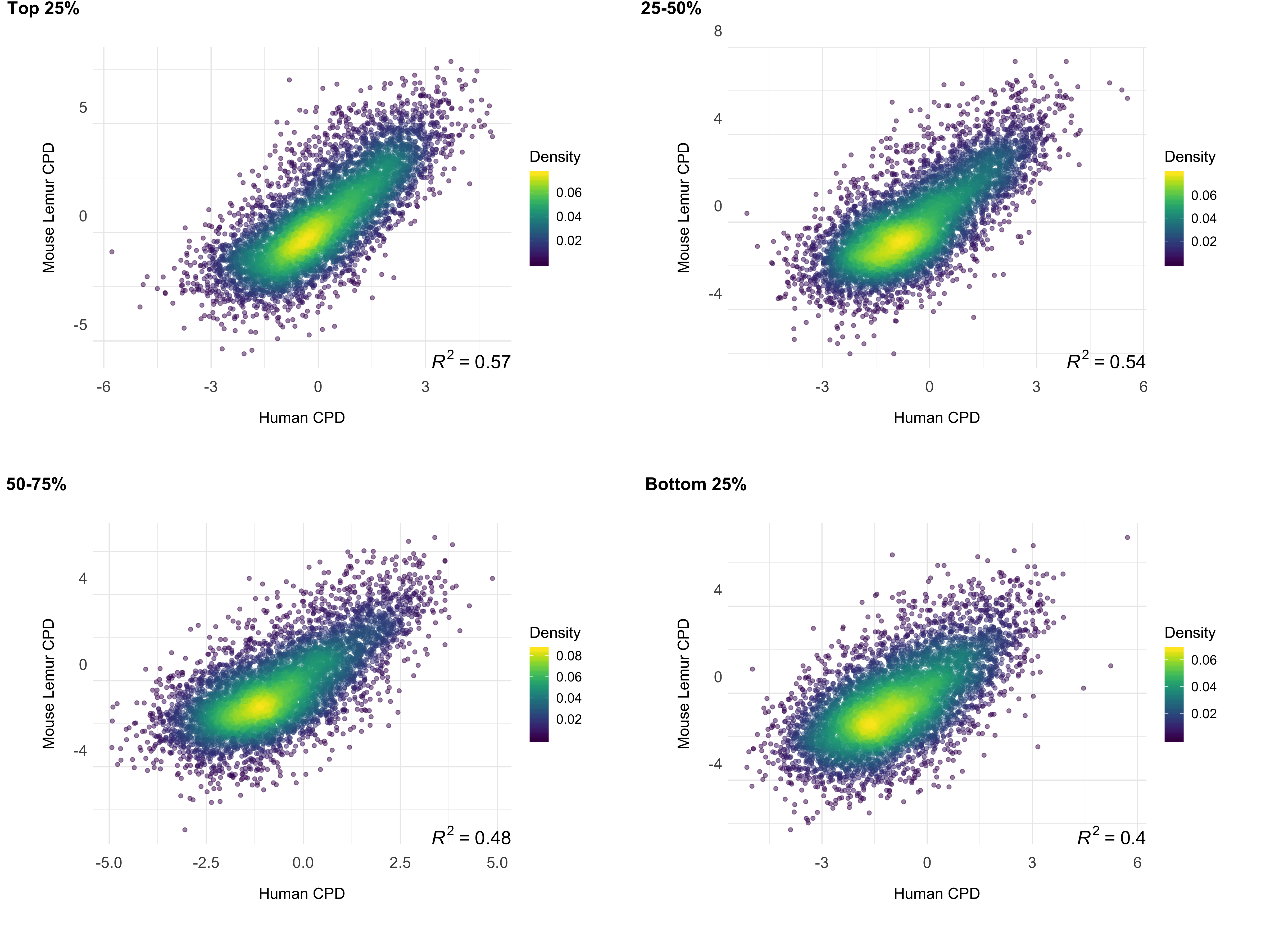


Figure S4: Human and mouse lemur CPD repair correlation in human transcription quartiles.


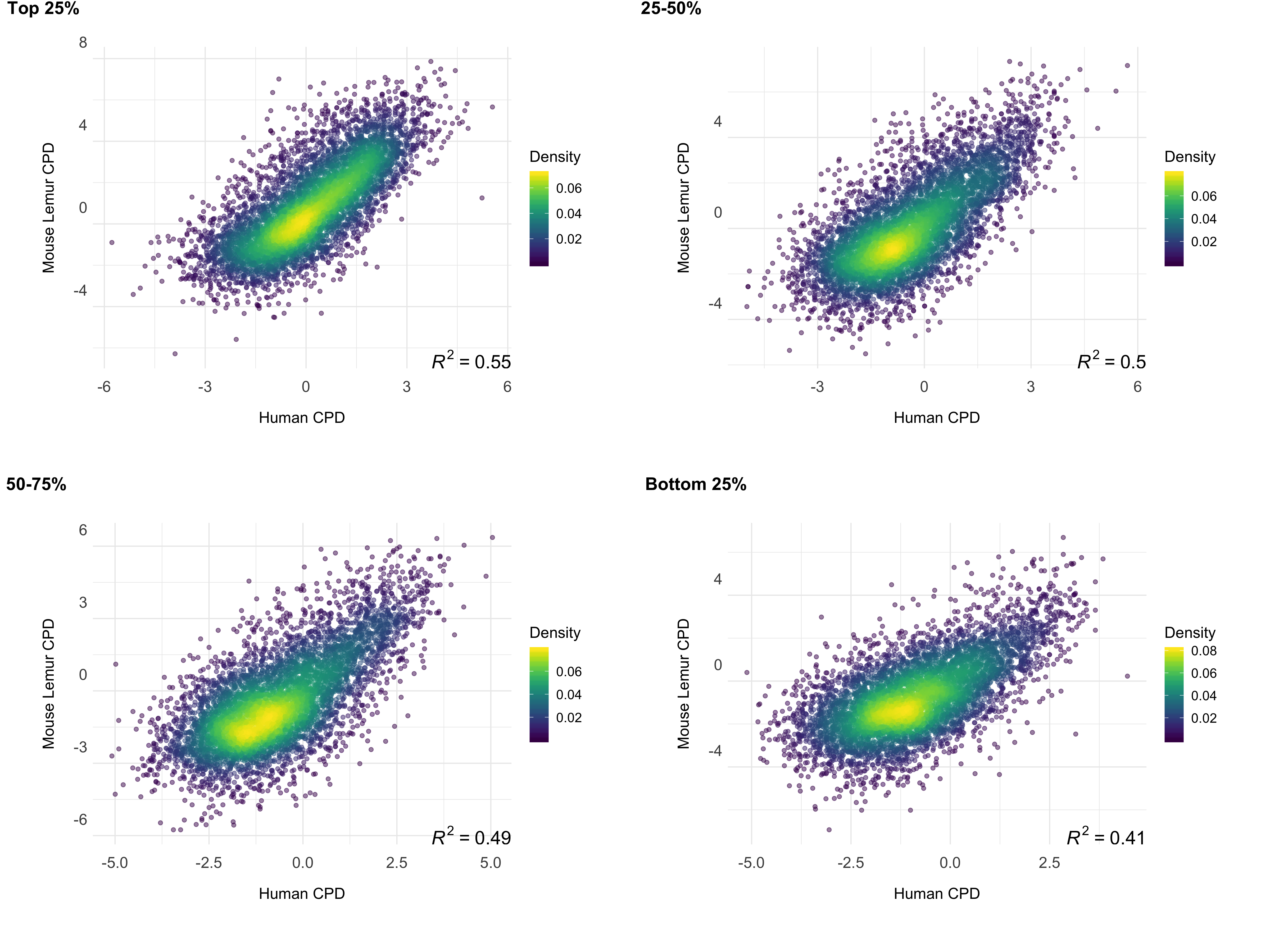


Figure S5: Human and mouse lemur CPD repair correlation in mouse lemur transcription quartiles.


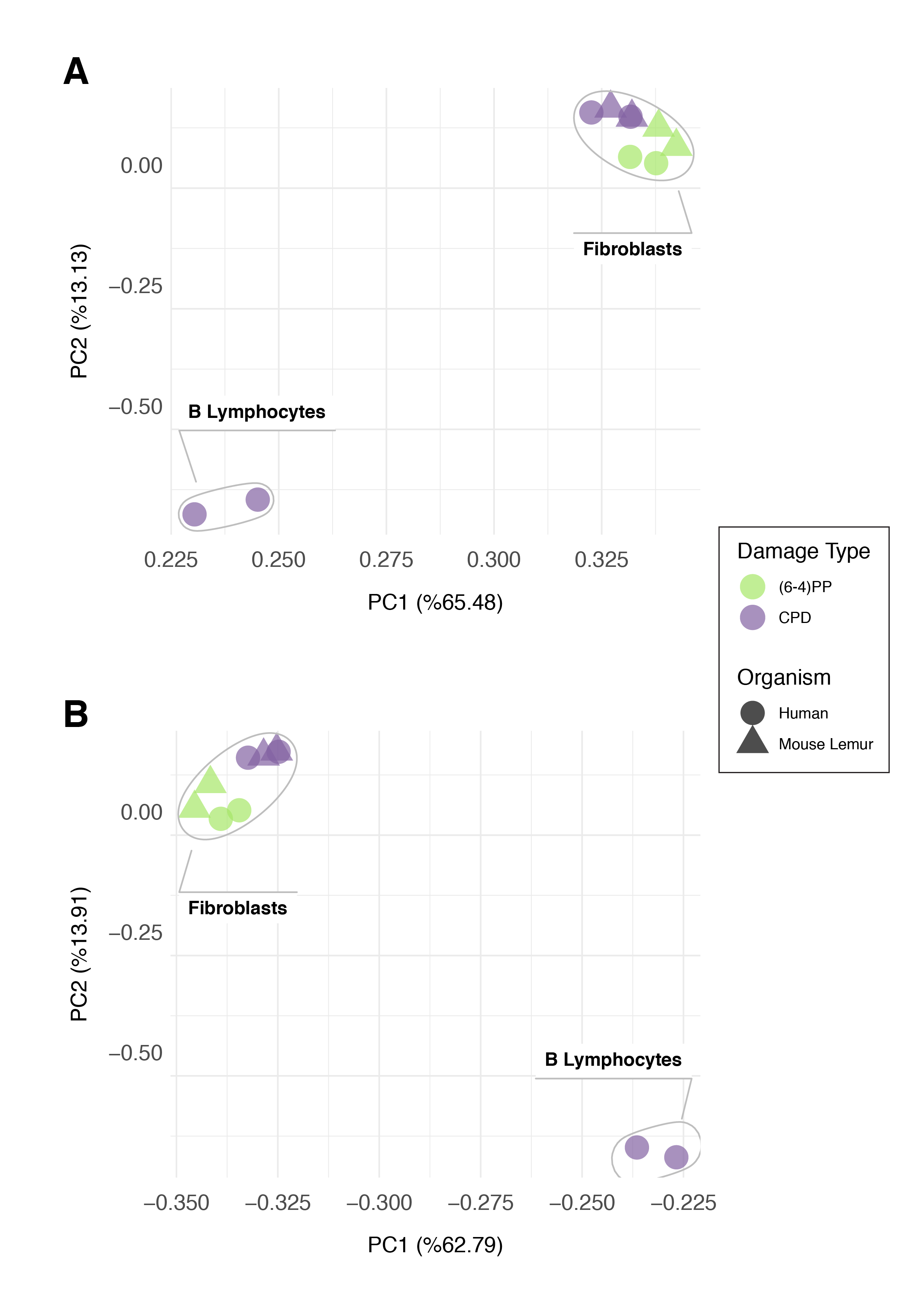


Figure S6: PCA plot for XR-seq reads mapped on the genic (A) and intergenic (B) regions.
